# Increased Aortic Exclusion in Endovascular Treatment of Complex Aortic Aneurysms †

**DOI:** 10.3390/jcm12154921

**Published:** 2023-07-26

**Authors:** Merel Verhagen, Daniel Eefting, Carla van Rijswijk, Rutger van der Meer, Jaap Hamming, Joost van der Vorst, Jan van Schaik

**Affiliations:** 1 Department of Vascular Surgery, Leiden University Medical Center, 2333 ZA Leiden, The Netherlands; m.j.verhagen@lumc.nl (M.V.); d.eefting@lumc.nl (D.E.); j.f.hamming@lumc.nl (J.H.); j.r.van_der_vorst@lumc.nl (J.v.d.V.); 2 Department of Radiology, Leiden University Medical Center, 2333 ZA Leiden, The Netherlands; c.s.p.van_rijswijk@lumc.nl (C.v.R.); r.w.van_der_meer@lumc.nl (R.v.d.M.)

**Keywords:** complex aortic aneurysm, endovascular repair, open repair, aortic exclusion, Crawford classification

## Abstract

Purpose: Perioperative risk assessments for complex aneurysms are based on the anatomical extent of the aneurysm and do not take the length of the aortic exclusion into account, as it was developed for open repair. Nevertheless, in the endovascular repair (ER) of complex aortic aneurysms, additional segments of healthy aorta are excluded compared with open repair (OR). The aim of this study was to assess differences in aortic exclusion between the ER and OR of complex aortic aneurysms, to subsequently assess the current classification for complex aneurysm repair. Methods: This retrospective observational study included patients that underwent complex endovascular aortic aneurysm repair by means of fenestrated endovascular aneurysm repair (FEVAR), fenestrated and branched EVAR (FBEVAR), or branched EVAR (BEVAR). The length of aortic exclusion and the number of patent segmental arteries were determined and compared per case in ER and hypothetical OR, using a Wilcoxon signed-rank test. Results: A total of 71 patients were included, who were treated with FEVAR (n = 44), FBEVAR (n = 8), or BEVAR (n = 19) for Crawford types I (n = 5), II (n = 7), III (n = 6), IV (n = 7), and V (n = 2) thoracoabdominal or juxtarenal (n = 44) aneurysms. There was a significant increase in the median exclusion of types I, II, III, IV, and juxtarenal aneurysms (*p* < 0.05) in ER, compared with hypothetical OR. The number of patent segmental arteries in the ER of type I–IV and juxtarenal aneurysms was significantly lower than in hypothetical OR (*p* < 0.05). Conclusion: There are significant differences in the length of aortic exclusion between ER and hypothetical OR, with the increased exclusion in ER resulting in a lower number of patent segmental arteries. The ER and OR of complex aortic aneurysms should be regarded as distinct modalities, and as each approach deserves a particular risk assessment, future efforts should focus on reporting on the extent of exclusion per treatment modality, to allow for appropriate comparison.

## 1. Introduction

The current classification for complex aortic aneurysms is based on the anatomical extent of the aneurysm. With the management of complex aortic aneurysms always having been associated with significant rates of adverse outcomes, the purpose of the original Crawford classification was to recognize differences in the intra- and postoperative risks of complications and mortality in the open repair of these aneurysms [1]. Based on the anatomical dimensions, aortic aneurysms were categorized in types I–IV, with Safi et al. later adding type V [2,3], which contributed greatly to standardized reporting in complex aortic surgery.

With the availability of endovascular repair for complex aortic aneurysms, management options have significantly increased as more frail patients can be considered for surgery [4,5]. The treatment modality offers therapeutic options for patients unfit for open surgery due to decreased cardio-pulmonary stress, blood loss, and surgical trauma [6,7]. However, endovascular treatment leads to an increase in aortic exclusion compared with open repair, as a result of additional segments of healthy aorta being sacrificed in order to ensure adequate proximal and distal seal [8,9,10]. As a consequence of the increased extent of aortic exclusion in endovascular repair, the Crawford classification, based on the anatomical extent of the aneurysm, might not provide for an accurate assessment of full aortic exclusion in endovascular repair. Imaginably, this could have significant consequences for reporting on complex aortic aneurysm repair, and the subsequent assessment of treatment options and prognostic risks. There is currently no widely adopted system to specify aortic exclusion in the endovascular repair of complex aortic aneurysms, resulting in a heterogeneity among methods used to report on the extent of treated aorta [11,12,13,14].

This study primarily aimed to evaluate differences in the length of aortic exclusion in the open and endovascular repair of complex aortic aneurysms, as well as differences in the loss of patent segmental arteries and treated visceral arteries, to subsequently reflect on the suitability of the current classification system in the endovascular era.

## 2. Materials and Methods

### 2.1. Study Design and Patient Cohort

A single center retrospective observational study was performed, which was presented to the Medical Ethics committee who waived the need for medical ethical approval under Dutch law. Patients that were primarily treated for a complex aortic aneurysm, by means of fenestrated endovascular aneurysm repair (FEVAR), combined fenestrated and branched EVAR (FBEVAR), or branched EVAR (BEVAR), at the department of Vascular Surgery between 2013 and 2020, were included in the study. Patients with connective tissue disease, as well as patients without a postoperative computed tomography angiography (CTA) follow up, were excluded. The primary outcomes of this study included the length of excluded aorta in hypothetical open and actual endovascular repair of complex aortic aneurysms, the number of patent segmental arteries, and the number of renal and visceral arteries that had to be treated in both treatment modalities.

### 2.2. Patient and Aneurysm Characteristics

Complex aortic aneurysms were categorized as juxtarenal aneurysms [15], or according to the Crawford classification in the case of thoracoabdominal aneurysms (TAAA), ranging from Crawford type I to type V [16]. Preoperative data on patient demographics, comorbidities, aneurysm characteristics, and postoperative data on early outcomes were collected. Retrospective analysis of 1 mm thin slice images of the preoperative, and the first postoperative, CTA was performed. Endovascular exclusion was determined by creating central luminal line reconstructions using 3-mensio vascular™ (Pie Medical Imaging, Maastricht, The Netherlands). The length of aortic exclusion in hypothetical open aortic repair was determined using the same central luminal line reconstructions, measuring the aorta between the hypothetical proximal clamping site and the aortic bifurcation. The hypothetical cross clamping location for an open approach was discussed and determined by two vascular surgeons (JS and JV). The length of the endovascular aortic exclusion was determined by measuring the aorta from the proximal covered seal of the stent graft up to the anatomical aortic bifurcation, as no segmental arteries originate from the common iliac artery. Patent segmental arteries were assessed by scoring the number of contrast-filled segmental arteries throughout the entire aorta, both pre- and postoperatively in ‘open’ and endovascular repair. Similarly, the number of treated visceral arteries was assessed by determining the number of visceral arteries that would need to be treated in ‘open repair’ (e.g., through clamping or reinsertion), and that were intraluminally manipulated in endovascular repair (e.g., through wire manipulation for visceral vessel stenting).

### 2.3. Perioperative Management

Patients were treated with custom-made or off-the-shelf endografts obtained from Cook Medical^®^ (Bloomington, IN, USA), Medtronic^©^ (Northridge, CA, USA), or Terumo Aortic^©^ (Inchinnan, UK). The type of device was selected according to the patients’ anatomy and urgency of the procedure. The endografts were designed according to the instructions for use (IFU) with intentional proximal and distal sealing zone lengths of at least 25 mm, taking into consideration the aortic diameter, mural thrombus, and eccentric wall dilatation. All elective procedures for TAAA were planned as staged procedures.

Patients were treated by a dedicated team of vascular surgeons and interventional radiologists, experienced in performing open and endovascular complex aortic repair. A standardized protocol was used to prevent the occurrence of spinal cord ischemia (SCI), consisting of spinal drainage and periprocedural neuromonitoring (e.g., motor-evoked potentials and somatosensory-evoked potentials). Carotid subclavian bypass was performed in all cases where proximal sealing necessitated coverage of the left subclavian artery. To provide for a durable distal seal, bi-iliac distal landing was performed in a substantial part of the patients. Postoperative management in TAAA repair consisted of spinal drainage during the first 24–72 h after the procedure. A mean arterial pressure (MAP) of 75 mmHg was maintained postoperatively, and hemoglobin was kept above 7 mmol/L. All patients included were followed up and underwent a CTA within 6 weeks after the (finalizing) procedure.

### 2.4. Statistical Analyses

Continuous data were reported as mean and standard deviation. Categorical data were presented as prevalence in the population by reporting absolute numbers and percentages. For aortic exclusion in open or endovascular approach, as well as for the number of patent segmental arteries, data were reported as median and interquartile ranges [IQR]. A Wilcoxon signed-rank test was used to compare the length of exclusion and patent segmental arteries in ‘open’ and endovascular repair. A *p*-value of < 0.05 was considered significant. Analyses were performed in collaboration with a medical statistician, using IBM SPSS software.

## 3. Results

Between May 2013 and September 2021, 74 patients underwent endovascular treatment of a complex aortic aneurysm, of which 71 patients were included in this study. Three patients were excluded due to the absence of a postoperative CTA, which was due to periprocedural mortality (n = 2) and following patients’ explicit request for follow-up with duplex ultrasound (n = 1). The mean age of the study population was 73 years (±6.1), with 81.7% being male (Table 1). There were five patients with a Crawford type I, seven with a type II, six with a type III, seven with a type IV, and two with a type V TAAA, and forty-four patients had a juxtarenal aneurysm. The mean maximal aneurysm diameter was 64.6 mm (±10 mm). A total of forty-four patients were treated by means of FEVAR (62%), eight by means of FBEVAR (11.2%), and nineteen with BEVAR (26.8%). There were three emergency procedures.

### 3.1. Aortic Exclusion in Open Versus Endovascular Repair

**Figure 1:**
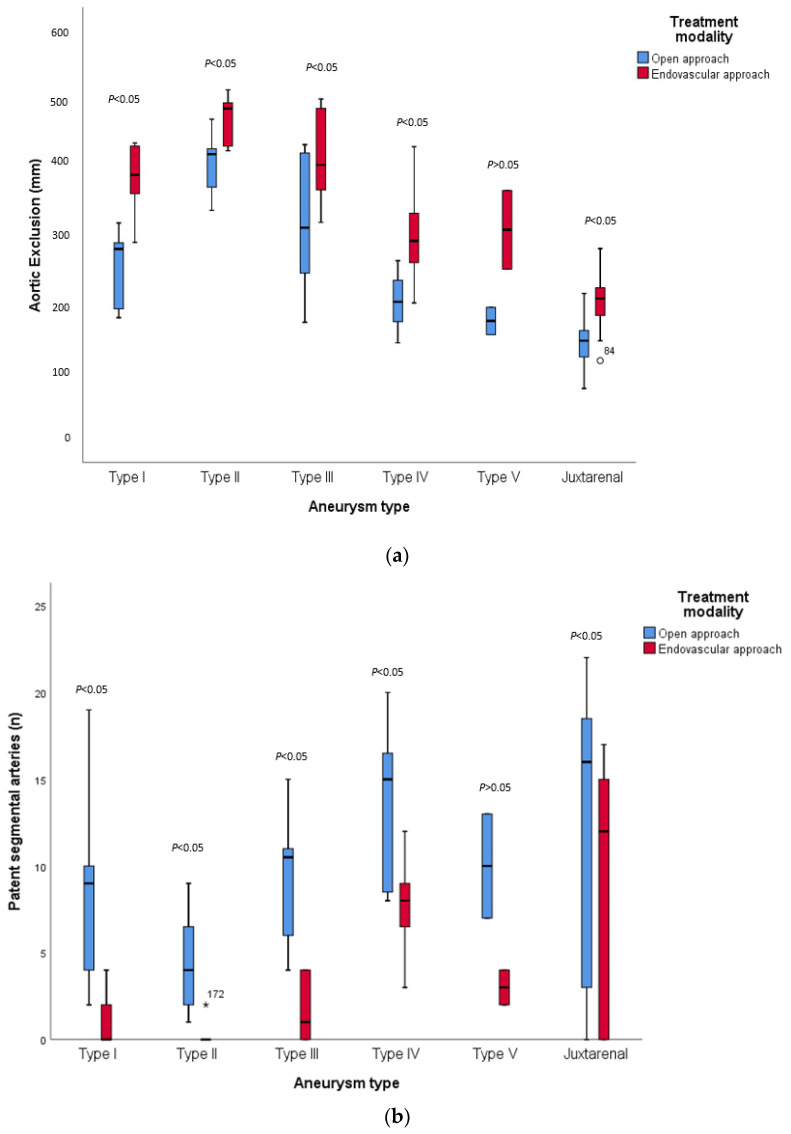
(**a**) differences in the median length of aortic exclusion in ‘open’ versus endovascular repair; (**b**) differences in patent segmental arteries in ‘open’ versus endovascular repair.

The median length of the excluded aorta in type I TAAAs was 279 mm [186, 303 mm] in ‘open’ versus 388 mm [325, 432 mm] in endovascular treatment (*p* < 0.05) (Figure 1a and Supplementary Table S1). For type II aneurysms, the median length was 418 mm [356, 434 mm] compared with 485 mm [425–498 mm] in ‘open’ and endovascular repair, respectively (*p* < 0.05). For type III aneurysms, ‘open’ treatment excluded a median length of 311 mm [226, 423 mm] compared with 403 mm [354, 489 mm] in endovascular repair (*p* < 0.05). The estimated length of exclusion was 202 mm [144, 259 mm] in ‘open’ repair versus 291 mm [244, 353 mm] in the endovascular repair of type IV aneurysms (*p* < 0.05) and 174 mm (28 mm) in ‘open’ compared with 308 mm (81 mm) in the endovascular repair of type V aneurysms (*p* > 0.05). For juxtarenal aneurysms, the median length in ‘open’ treatment was 145 mm [121, 161 mm] versus 207 mm [182, 223 mm] in endovascular repair (*p* < 0.05).

### 3.2. Patent Segmental Arteries in Open and Endovascular Repair

For Crawford type I aneurysms, in hypothetical open repair, a median of 9 [3, 14.5] patent segmental arteries would remain (Figure 1b and Supplementary Table S1), compared with 0 [0, 3] patent segmental arteries in endovascular treatment (*p* < 0.05). There were a median of 4 [2, 7] patent segmental arteries in ‘open’ versus 0 [0, 0] in endovascular repair of type II aneurysms (*p* < 0.05), and 10.5 [5.5, 12] versus 1 [0, 4] in type III aneurysms.

For the type IV TAAAs, there were 15 [8,9,10,11,12,13,14,15,16,17] patent segmental arteries in ‘open’ treatment, compared with 8 [5, 10] segmental arteries in endovascular repair (*p* < 0.05). A mean of 10 (4.2) segmental arteries were patent in hypothetical open repair versus 3 (1.4) in endovascular repair (*p* > 0.05). Lastly, there were a median of 16 [2.5, 18.75] patent segmental arteries in the ‘open’ repair of juxtarenal aneurysms, compared with 12 [0, 15] in endovascular repair (*p* < 0.05).

### 3.3. Treated Visceral Arteries

In Crawford type I, II, III, IV, and V aortic aneurysms, there is no difference in the number of treated visceral arteries in ‘open’ or endovascular repair as all four are necessarily included in the repair (Supplementary Table S1). In the ‘open’ repair of juxtarenal aneurysms, four visceral arteries would have to be treated in one case (n = 1; 2.3% of all cases), two arteries in sixteen cases (n = 16; 36.3%), one artery in eight cases (n = 8, 18.2%), and zero arteries in the ‘open’ repair of nineteen cases (n = 19; 43.2%), averaging one visceral vessel per case. In the endovascular repair of these juxtarenal aneurysms, there were four treated arteries in ten cases (n = 10; 22.7%), three visceral arteries in twenty four cases (n = 24; 54.5%), two arteries in six cases (n = 6; 13.6%), and one artery in four cases (n = 4; 9.1%), averaging 2.9 visceral vessels per case.

## 4. Discussion

This study identified significant differences in the length of aortic exclusion between endovascular and hypothetical open treatment of TAA and juxtarenal aneurysms. Increased exclusion in endovascular repair inadvertently resulted in a lower number of patent intercostal and lumbar arteries. The endovascular treatment of juxtarenal aneurysms led to a higher number of treated visceral arteries, compared with open repair.

**Figure 2:**
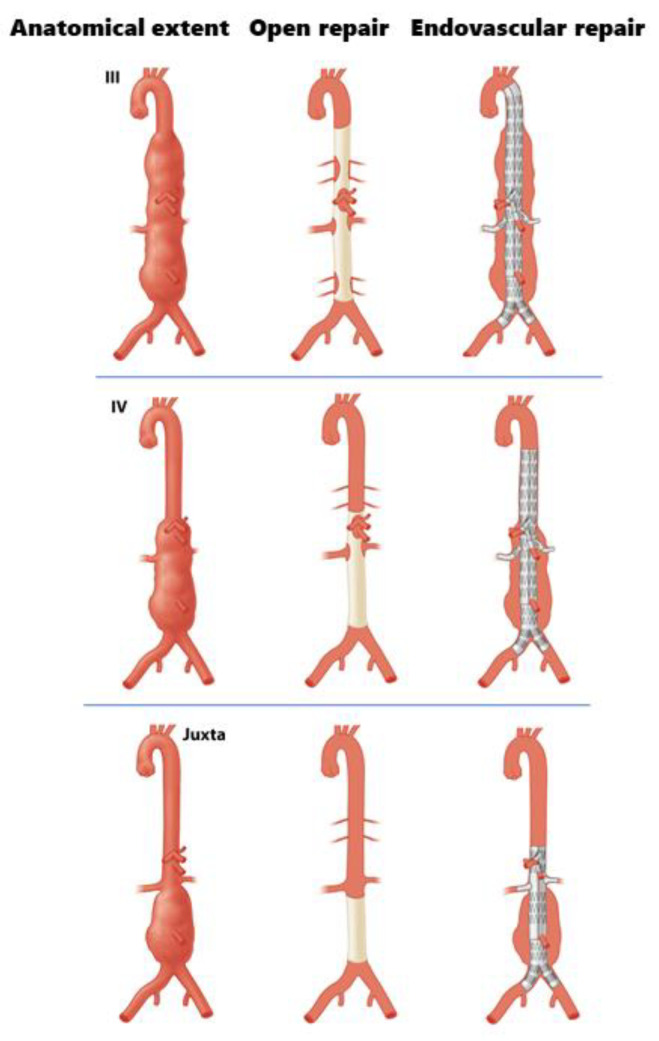
Examples of differences in anatomical extent and aortic exclusion in open and endovascular repair of three types of complex aortic aneurysms.

**Figure 3:**
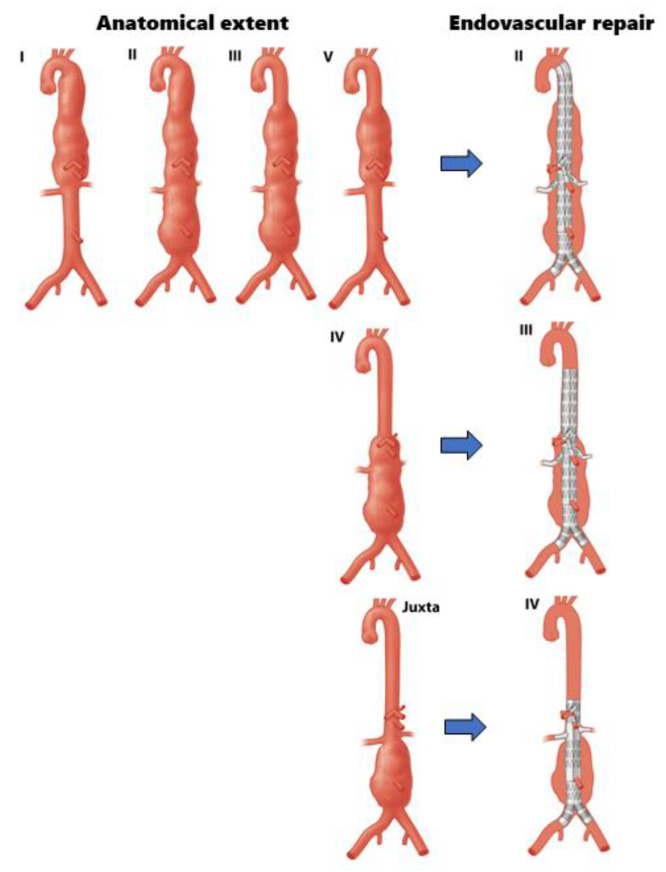
Illustration of how endovascular treatment of TAA and juxtarenal aneurysms may lead to a length of aortic exclusion that is comparable to the (anatomical) extent of Crawford types II, III, and IV.

Based on our results, it can be concluded that the length of aortic tissue treated endovascularly is not comparable to the original anatomical extent of a complex aortic aneurysm, which has traditionally formed the basis for the Crawford classification. Examples of differences between the anatomical extent of an aneurysm, the extent of aortic repair in open, and the length of aortic exclusion in endovascular repair are illustrated in Figure 2. It could be argued that, when assessing the extent of aortic exclusion in endovascular repair for different types of complex aortic aneurysms more closely, the endovascular length of exclusion matches the anatomical extent of a different Crawford type. For instance, when performing endovascular repair of a juxtarenal aneurysm, the extent of this repair, which may often require four fenestrations, matches the anatomical extent of a Crawford type IV (Figure 3). Similarly, when using FEVAR to repair a Crawford type IV, the proximal seal might result in aortic exclusion matching the anatomical extent of a Crawford type III. Imaginably, this makes it questionable whether the clinical outcomes of open and endovascular repair of a Crawford type IV TAAA can be compared at all, as both treatment modalities consider widely varying lengths of aortic exclusion.

Our results are supported by recent work by Oderich et al. that underlines the importance of reporting on the extent of aortic exclusion in the endovascular repair of thoracoabdominal aneurysms, since the added seal for stent grafts differs from the would-be surgical anastomosis [8]. Oderich et al. recommend using a numerical system to indicate zones required for endovascular treatment and to calculate the estimated segments covered as a result of aortic exclusion to ultimately facilitate proper reporting on outcome and risk assessment, thereby facilitating comparison and benchmarking. Our study confirms this theoretical concept in an observational clinical setting: when comparing the zones required for the anastomosis in open repair or the sealing in endovascular repair for different types of complex aortic aneurysms according to the numerical system, similar correlations between the extent of open and endovascular exclusion are found. Another idea could be to revise the traditional Crawford classification, to make it applicable to both treatment modalities, by differentiating between conventional O-Crawford (for open repair) and E-Crawford (for endovascular repair). This way, as illustrated in Figure 3, an O-Crawford type IV would be considered an E-Crawford type III.

Three other studies have focused on differences in aneurysm extent and aortic exclusion in complex endovascular aortic treatment. A study by Feezor et al. centered on thoracic endovascular repair, by identifying the length of a thoracic EVAR (TEVAR) graft as a significant risk factor for the incidence of SCI [17]. Gallitto et al. focused on custom-made FEVAR, portraying a mean additional aortic coverage of 48 mm proximally in juxta-, pararenal aneurysms and type IV TAAAs, with no significant effect on clinical outcomes [12]. Most segmental arteries were sacrificed in the repair of type IV aneurysms. These results align with our findings that, in accordance with a relatively small increase in aortic exclusion as a result of FEVAR instead of open repair, few segmental arteries were sacrificed. The difference in treated visceral arteries was not discussed. Lastly, Bertoglio et al. reported a greater sacrifice of healthy aortic tissue and intercostal arteries in TAAAs treated with an off-the-shelf branched device, compared with open repair [11]. As these devices nearly always require an additional proximal thoracic stent, these results cannot be compared to cases treated with custom-made branched devices. Further research should focus on a comparison of stent types and design strategies.

In the treatment of complex aortic aneurysms, a dilemma may arise between providing for a durable treatment and the increased risk of SCI, as a result of pursuing the IFUs or adjusting for anatomical aspects of the aneurysm. Imaginably, increased aortic exclusion entails an increased risk for SCI, yet the incidence of this complication depends on many other risk factors as well [18]. It is worthwhile appreciating the large disparities in the open and endovascular treatment of complex aneurysms. For instance, open repair includes the option of the reimplantation of segmental arteries, which is not possible in endovascular treatment. On the other hand, in open repair there is the postoperative risk of para-anastomotic aneurysm development, especially in the case of a proximal anastomosis close to the healthy aorta [19]. Also, open repair is more frequently associated with intra-, and postoperative systemic hypoperfusion due to blood loss or a more severe systemic inflammatory response, in turn increasing the risk of SCI [20,21]. Endovascular repair allows for staged treatment, possibly stimulating the collateral recruitment of spinal perfusion [18,22,23]. The current literature is not conclusive as of yet, with studies that report long proximal landing zones in fenestrated and branched EVAR resulting in low rates of SCI [24,25,26]. This is opposed to other studies that identified a relation between fenestrated grafts with a coverage of over 52 mm above the celiac artery and an increased risk of SCI [13]. As a result, the clinical consequence of preserving, for instance, 16 segmental arteries in the open, compared with 12 in the endovascular, repair of juxtarenal aneurysms, as was found in this study, is unknown.

Regarding the association between aortic exclusion and the risk of visceral complications in open or endovascular repair, various aspects are of influence, such as intra- or extraluminal manipulation of visceral or renal arteries. Endovascular treatment encompassing multiple fenestrations or branches can be demanding as it implies extensive intraluminal wire manipulation [27], whereas in open repair of TAAA, the selective perfusion of visceral and renal arteries is the golden standard. Imaginably, wall thrombus or irregular aortic diameters may lead to the application of three or four fenestrations in the case of juxtarenal aneurysm repair, as opposed to suprarenal clamping alone in open repair. A tendency to apply an increased number of fenestrations in the endovascular repair of aneurysms with similar anatomy over time has been described in experienced centers, illustrating how the complexity of the devices is evolving [13]. Despite extensive endovascular repair being safe, there have been concerns about more fenestrations increasing the risks for, amongst others, visceral complications, apart from the known prolonged operating and fluoroscopy time [9,27]. This is supported by a series of 610 patients undergoing endovascular repair for a juxtarenal or thoracoabdominal aneurysm, in which Mastracci et al. found that, with the increasing complexity of the devices, there was an accompanying increase in celiac occlusions and the need for reinterventions [13].

As for type V TAAAs and juxtarenal aneurysms, it should be noted that open repair is still a treatment to consider when looking at durability and long-term outcomes [21,28]. Work by Michel et al., for instance, has shown open repair to be cost-effective, compared with endovascular repair with F-/B-EVAR [29]. To conclude, taking into consideration how the risks of complications, such as SCI or visceral occlusions, are influenced by many different factors, as well as the aspects of durability and costs, open and endovascular treatments should be regarded as distinct modalities, each with their particular risk assessment.

### Limitations

As this study was primarily intended to assess differences in aortic exclusion, in the number of patent segmental arteries and in the number of visceral arteries treated for both open and endovascular repair, a statistical assessment of the relation between aortic exclusion and clinical outcomes per Crawford type was beyond the scope of this study. To compare treatment modalities, a case-matched analysis of endovascular and open treatment of TAAAs was discussed but was deemed unreliable, due to the often unique anatomical features of complex aneurysms and heterogenous patient characteristics. This study served as the first exploration of an idea, and estimating hypothetical open repair was considered a suitable method for serving our research goal. Yet, the results of our study could be limited by the subjectivity of the assessment of the hypothetical open repair, as well as by the possibility of a difference in the intended clamp position and the eventual anastomosis (e.g., which changed as a result of anatomical factors perioperatively). Nevertheless, the extent of open repair was determined simultaneously by two vascular surgeons, experienced in the open and endovascular treatment of TAAAs, as this would normally be decided upon during a preoperative multidisciplinary team meeting. As the option to reimplant segmental arteries in the open repair of Crawford type I, II and III aneurysms is decided on perioperatively, this was not included in the assessment of patent segmental arteries in hypothetical open repair. Also, it should be noted that in the beginning of the complex aortic program at our hospital, 2-FEVAR procedures were performed, whereas today, according to advancing insights, these repairs are avoided. Nevertheless, data on 2-FEVARS were included in the data. Finally, the statistical power of the results is limited by the small groups of patients per Crawford type. This relates specifically to the type V TAAAs, of which a limited number of cases were included.

## 5. Conclusions

There are significant differences in lengths of aortic exclusion, patent segmental arteries, and the number of visceral arteries treated between the endovascular and hypothetical open repair of complex aortic aneurysms. The anatomical extent of these aneurysms does not match the length of aortic exclusion in endovascular repair, which might limit the suitability of a classification and subsequent risk assessment that was originally meant for an open repair of TAAA. Considering the differences in operation technique and the length of aortic exclusion, endovascular and open treatment of complex aneurysms should be considered as distinct treatment modalities. Future efforts should focus on uniformity in reporting on the extent of exclusion per treatment strategy, to further explore the consequences of these differences for clinical outcomes.

## Figures and Tables

**Table 1:** Characteristics of included patients treated by means of FEVAR, FBEVAR, or BEVAR for a complex aortic aneurysm.

Characteristics	Title 2
Patients (n)	71
Aneurysm extent (n) Crawford type I Crawford type II Crawford type III	5 7 6
Crawford type IV	7
Crawford type V	2
Juxtarenal	44
Maximal aortic aneurysm diameter (mm) (mean, SD)	64.6 (10.0)
Aneurysm etiology (%)	
Post-dissection aneurysm	2.8
Atherosclerosis	93
Unknown	4.2
Procedures (n, %)	71
FEVAR 1 or 2 fenestrations 3 or 4 fenestrations FBEVAR BEVAR 1 or 2 branches 3 branches 4 branches Priority (n, % emergency) Postoperative complications (%) Renal complications Temporary Permanent Intestinal ischemia Spinal cord ischemia 30-day mortality (%)	44 (62) 9 35 8 (11.2) 19 (26.8) 0 5 14 3 (4.2) 18.3 15.5 2.8 4.2 8.5 4.2

## Data Availability

Not applicable.

## Supplementary Materials

The following supporting information can be downloaded at: https://www.mdpi.com/article/10.3390/jcm12154921/s1, Supplementary Table S1. Length of aortic exclusion, number of patent segmental arteries and the number of manipulated visceral arteries in hypothetical open repair and endovascular complex aortic aneurysm repair.
