# Supplementary material for: Increased Aortic Exclusion in Endovascular Treatment of Complex Aortic Aneurysms
Source: J Clin Med. 2023 Jul 26;12(15):4921. doi: 10.3390/jcm12154921 (PMC10420108; doi:10.3390/jcm12154921)
Supplement: Supplementary file 1 [file jcm-12-04921-s001.zip › jcm-2464387-supplementary.pdf]

Supplementary Table S1. Length of aortic exclusion, number of patent segmental arteries and the number of manipulated visceral arteries in hypothetical open repair and endovascular complex aortic aneurysm repair.

	Estimated aortic exclusion in 'open' repair (mm) [median, IQR]	Aortic exclusion in endo-vascular repair (mm) [median, IQR]	Difference (mm) [median, IQR]	*P-value*	Patent segmental arteries in 'open' repair [median, IQR]	Patent segmental arteries in endo-vascular repair [median, IQR]	*P-value*	Treated visceral arteries, 'open' repair, n (%)	Treated visceral arteries, endo-vascular repair
Crawford type I<br><br>(n=5)	279.4 [185.5, 302.9]	388 [324.7, 432.2]	97 [75.8, 198.5]	0.043	9 [3, 14.5]	0 [0, 3]	0.043	4 arteries, n=5 (100)	4 arteries, n=5 (100)
Crawford type II<br><br>(n=7)	418.2 [356.4, 434.1]	485 [425.1, 498.1]	66.8 [43, 87]	0.018	4 [2, 7]	0 [0, 0]	0.028	4 arteries, n=7 (100)	3 arteries, n=3* (42.9)<br><br>4 arteries, n=4 (57.1)
Crawford type III<br><br>(n=6)	310.5 [226, 423]	402.5 [354, 488.5]	106.6 [47.8, 153.9]	0.046	10.5 [5.5, 12]	1 [0, 4]	0.042	4 arteries, n=6 (100)	3 arteries*, n=2 (33)<br><br>4 arteries, n=4 (67)
Crawford type IV<br><br>(n=7)	202 [144.1, 259.1]	291.3 [244, 353.4]	91.1 [56.3, 149.3]	0.018	15 [8, 17]	8 [5, 10]	0.027	4 arteries, n=7 (100)	4 arteries, n=7 (100)
Crawford type V<br><br>(n=2)	174 (28.2)*	307,5 (81.3)*	133,4 (53.1)*	0.18	10 (4.2)	3 (1,4)	0.18	4 arteries, n=2 (100%)	4 arteries, n=2 (100%)
Juxtarenal<br><br>(n=44)	145.1[121 .2, 160.5]	207 [182.1, 222.6]	64 [48, 74.7]	0.000	16 [2.5, 18.75]	12 [0, 15]	0.000	4 arteries, n=1 (2.3)<br><br>3 arteries, n=0 (0)<br><br>2 arteries, n=16 (36.4)<br><br>1 artery, n=8 (18.2)<br><br>0 arteries, n=19 (43.2)	4 arteries, n=10 (22.7)<br><br>3 arteries, n=24 (54.5)<br><br>2 arteries, n=6 (13.6)<br><br>1 artery, n=4 (9.1)<br><br>0 arteries, n=0

*Pre-existent occluded coeliac trunk or renal artery.
